# Early diaphragm dysfunction assessed by ultrasonography after cardiac surgery: a retrospective cohort study

**DOI:** 10.3389/fcvm.2024.1457412

**Published:** 2024-10-09

**Authors:** Hongbo Huai, Min Ge, Zhigang Zhao, Ping Xiong, Wenjun Hong, Zhongli Jiang, Jianming Wang

**Affiliations:** ^1^ Department of Epidemiology, Center for Global Health, School of Public Health, Nanjing Medical University, Nanjing, China; ^2^ Department of Rehabilitation Medicine, Nanjing Drum Tower Hospital, The Affiliated Hospital of Nanjing University Medical School, Nanjing, China; ^3^ Department of Cardiac-Thoracic Surgery, Nanjing Drum Tower Hospital, The Affiliated Hospital of Nanjing University Medical School, Nanjing, China; ^4^ Department of Rehabilitation Medicine, The First Affiliated Hospital of Nanjing Medical University, Nanjing, China; ^5^ Department of Epidemiology, Key Laboratory of Public Health Safety and Emergency Prevention and Control Technology of Higher Education Institutions in Jiangsu Province, School of Public Health, Nanjing Medical University, Nanjing, China

**Keywords:** diaphragmatic dysfunction, cardiac surgery, noninvasive ventilation, oxygen supply support, hospital stay, risk factors, thoracic drainage, prognosis

## Abstract

**Objective:**

Approximately 10%–70% of patients may develop diaphragmatic dysfunction after cardiac surgery, which may lead to delayed weaning from mechanical ventilation, increased ICU stays, postoperative hospitalization stays, and respiratory complications. However, its impact on prognosis and risk factors remain controversy. Therefore, we conducted a retrospective cohort study in which we evaluated diaphragmatic dysfunction in patients who underwent cardiac surgery via bedside diaphragm ultrasound to investigate its prognosis and possible risk factors.

**Methods:**

Data from the electronic medical records system included case records and ultrasound images of the diaphragm for 177 consecutive patients admitted to the ICU following cardiac thoracotomy surgeries performed between June and September 2020. Diaphragmatic dysfunction was defined as a diaphragmatic excursion of less than 9 mm in women and less than 10 mm in men at rest, with an average thickening fraction of less than 20%. SPSS 25.0 software was used to analyse the relationships between patients' general information, intraoperative and postoperative factors and diaphragmatic dysfunction, as well as the impact on patients' hospitalization days, mechanical ventilation time and respiratory system complications.

**Results:**

The incidence of early postoperative diaphragmatic dysfunction after cardiac surgery was 40.7%. Patients with diaphragmatic insufficiency were more likely to sequentially use noninvasive ventilation within 24 h after weaning off mechanical ventilation (3.8% vs. 12.5%, *P* = 0.029) and to require more oxygen support (23.8% vs. 40.3%, *P* = 0.019). Although there was no significant difference, the diaphragmatic dysfunction group tended to have longer ICU stays and postoperative hospital stays than did the normal diaphragmatic function group (*P* = 0.119, *P* = 0.073). Univariate and multivariate logistic regression analyses both revealed that chest tube drainage placed during surgery accompanied by bloody drainage fluid was an independent risk factor for diaphragmatic dysfunction (univariate analysis: 95% CI: 1.126–4.137, *P* = 0.021; multivariate analysis: 95% CI: 1.036–3.897, *P* = 0.039).

**Conclusion:**

Eearly diaphragmatic dysfunction after cardiac surgery increased the proportion of patients who underwent sequential noninvasive ventilation after weaning from mechanical ventilation and who required more oxygen. Chest tube drainage placed during surgery accompanied by bloody drainage fluid was an independent risk factor for diaphragmatic dysfunction, providing evidence-based guidance for respiratory rehabilitation after cardiac surgery.

## Introduction

The diaphragm is the primary inspiratory muscle, generating 70% of the inspiratory force. According to the literature, approximately 10%–70% of patients who undergo cardiac surgery experience diaphragmatic dysfunction (1). Diaphragmatic fiber weakness develops due to a reduction in diaphragmatic contractile proteins and the activation of proteolytic pathways two hours after thoracic surgery (2). Diaphragmatic ultrasound has recently emerged as a widely used noninvasive method for evaluating diaphragm function (3, 4). It offers advantages such as ease and affordability of operation, absence of ionizing radiation, noninvasiveness, and high reproducibility. This method enables accurate and dynamic assessment of diaphragm movement and conformation (3, 5–7).

Previous studies have suggested that diaphragmatic dysfunction after cardiac surgery has a detrimental effect on prognosis, leading to insufficient ventilation, nighttime sleep disorders, pneumonia, atelectasis, and an increased incidence of reintubation. This could prolong the duration of mechanical ventilation in critically ill patients and increase their length of stay in the ICU (5, 8–11). However, some scholars have reported that unilateral diaphragmatic dysfunction does not increase the ICU length of stay, mechanical ventilation time, or incidence of respiratory complications (12, 13). Therefore, the impact of diaphragmatic dysfunction on prognosis needs to be reevaluated. In addition, the specific causes and mechanisms of early diaphragm dysfunction following cardiac surgery remain unclear. Possible causes include phrenic nerve frostbite due to the use of frozen perfusion fluid for myocardial protection, the impaired blood supply to the phrenic nerve due to internal mammary artery ligation, diaphragm atrophy following thoracotomy, mitochondrial hyperoxidative stress, increased release of proinflammatory factors, prolonged mechanical ventilation, and multiple organ failure. A reduced diaphragmatic cross-sectional area and disuse atrophy can result in diaphragmatic dysfunction (2, 3, 5, 8, 9, 14). However, there is still debate over whether certain risk factors, such as cardiopulmonary bypass duration, coronary bypass surgery, obesity, and local hypothermia, can lead to diaphragm dysfunction (8, 12, 15).

Early intervention for diaphragmatic dysfunction can reduce in-hospital mortality. For patients with diaphragmatic dysfunction, certain treatments can facilitate the recovery of diaphragmatic function (16–18). Therefore, timely detection of diaphragm dysfunction is crucial for patient prognosis and treatment. To date, few studies have employed bedside ultrasound to investigate the risk factors for early postoperative diaphragmatic dysfunction and its influence on prognosis. Additionally, differing perspectives regarding its effects on patient outcomes and the associated risk factors necessitate further investigation. Therefore, we conducted a retrospective cross-sectional study evaluating diaphragmatic dysfunction in patients after cardiac surgery via bedside diaphragm ultrasound to investigate its prognosis and possible risk factors.

## Materials and methods

### Ethics statement

This study was approved by the Medical Ethics Committee of Nanjing Drum Tower Hospital (Ethics Number: 2022-425-01) and registered at the China Clinical Trial Registration Centre (Registration Number: ChiCTR2300076339, https://www.chictr.org.cn). Informed consent was obtained from all the subjects.

### Study design and participants

This was a retrospective cohort study. The participants in this study were patients who underwent cardiothoracic surgery at Nanjing Drum Tower Hospital between June and September 2020 and were subsequently admitted to the intensive care unit (ICU). These patients underwent bedside ultrasound examination of the diaphragm between 24 and 48 h postsurgery. The inclusion criteria were as follows: cardiopulmonary bypass surgery with median thoracotomy for the first time, including valve replacement, coronary artery bypass surgery, or ascending aortic surgery; spontaneous breathing; bedside diaphragmatic ultrasound (Clover 60, Shenzhen Huasheng Medical Technology Co., Ltd.) performed within 24–48 h postoperatively, with clear and research-quality images (see the following diaphragm function assessment criteria); and aged >18 years. The exclusion criteria were as follows: severe complications, including brain dysfunction, Glasgow score ≤8, hemiplegia or paraplegia, or confirmed stroke within 72 h postoperatively; renal failure necessitating continuous blood purification therapy; severe heart failure or cardiogenic shock requiring high-dose vasoactive drugs or mechanical support; sternal not sutured; pregnancy or recent delivery; preoperative thymoma with myasthenia gravis; preoperative brain dysfunction from any cause; spinal cord injury (above C5) or phrenic nerve injury; and prior history of thoracotomy.

**Figure 1:**
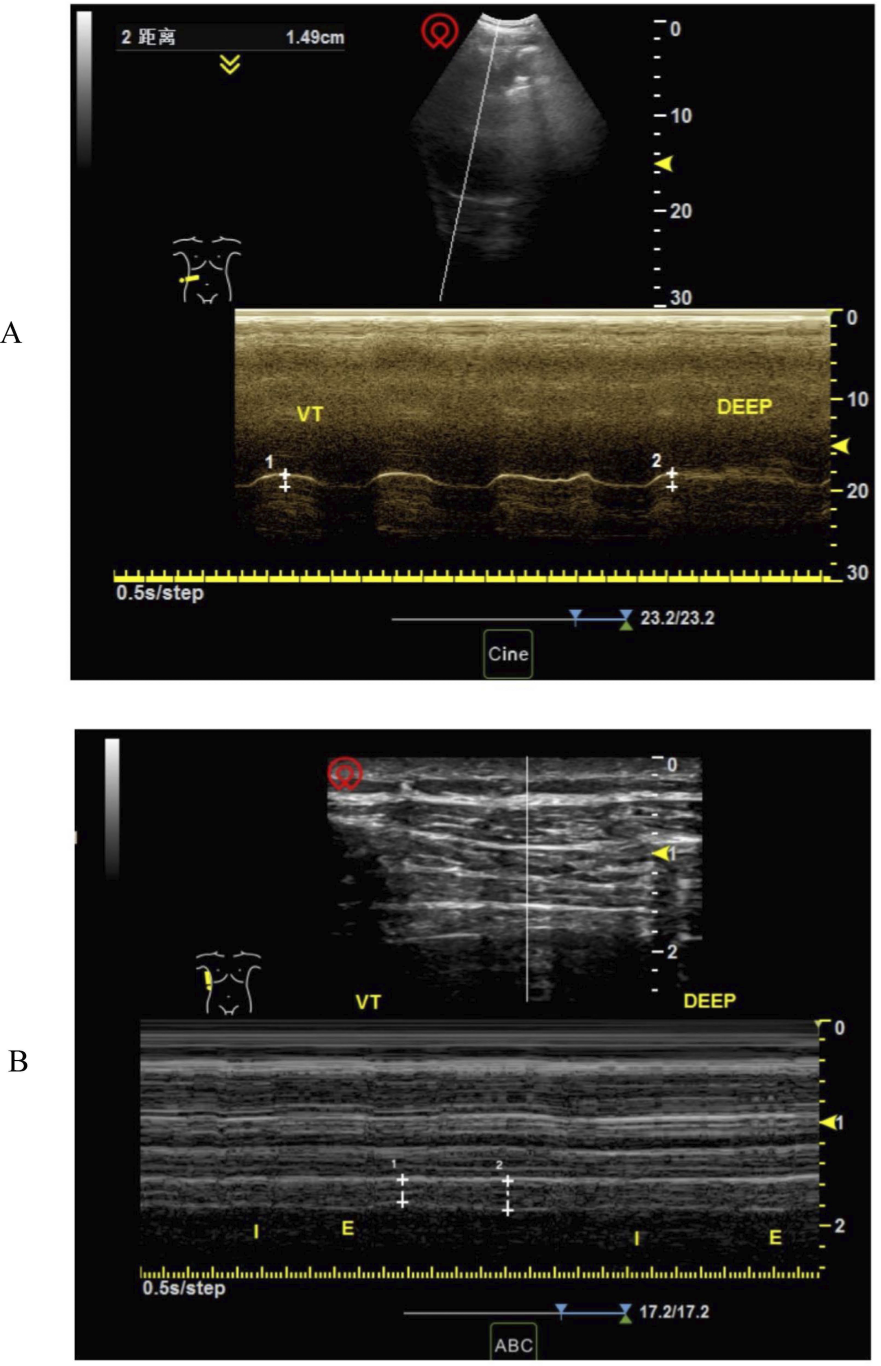
Measurement of right diaphragm excursion and thickness at rest. **(A)** Measurement of right diaphragm excursion at rest **(B)** measurement of diaphragm thickness at rest.

The diaphragmatic pictures of the screened patients met the following criteria: bilateral diaphragmatic activity and bilateral diaphragmatic thickness could be clearly measured, and three continuous respiratory cycles could be shown in a single picture. Diaphragmatic activity was measured during breathing at rest because postoperative pain, sternotomy, and other factors might affect the patient's effort during forced breathing or sniffing (12). The details of the measurements were as follows. To measure diaphragmatic activity, the probe was positioned at the inferior margin of the costal arch to observe diaphragmatic activity through the acoustic window of the liver or spleen. The distance between the end of inspiration and expiration was taken in the M mode as the diaphragmatic excursion (Figure 1). The average diaphragmatic activity was then calculated over three consecutive respiratory cycles. To determine the fraction of diaphragmatic thickness, the probe was placed perpendicularly to the chest wall at the mid-axillary line (T6-8) until the three-layer structure (pleura-diaphragm-peritoneum) could be observed under the B mode. The diaphragmatic thickness at the end of inspiration and expiration was calculated in M mode (Figure 1). The diaphragmatic thickness fraction was calculated as (end-inspiratory thickness to end-expiratory thickness)/end-expiratory thickness × 100%, and the average value of three consecutive respiratory cycles was calculated. On the basis of previous literature, diaphragmatic dysfunction in this study was defined as resting diaphragmatic excursion of less than 9 mm for women and less than 10 mm for men (8, 19, 20), and the average diaphragm thickness fraction was less than 20% (5, 9). Consequently, the participants were categorized into a normal diaphragm function group (Normal group) and a diaphragm dysfunction group (DD group).

### Data collection

We collected baseline data from the electronic case system, including sex, age, body mass index (BMI), comorbidities (hypertension and diabetes), preoperative information (preoperative NYHA classification of cardiac function), operation information (cardiopulmonary bypass time, CPB), surgical procedure information (valve replacement, cardiac bypass, off-pump heart bypass, ascending aorta surgery), placement of thoracic drainage tubes, and postoperative information, such as left ventricular ejection fraction (LVEF), Sequential Organ Failure Assessment (SOFA) score within 24 h after the operation, C-reactive protein (CRP) within 24 h after the operation), blood loss, blood transfusion, the use of sedatives (propofol or dextromethorphan), analgesics (morphine, fentanyl or remifentanil), and bloody drainage fluid from the thoracic drainage tube. The primary outcomes in this study were the incidence of postoperative pulmonary complications, such as reintubation, tracheostomy, and noninvasive ventilation; the incidence of hypoxemia (oxygen partial pressure < 80 mmHg); and the oxygen supply level within 24 to 48 h after surgery within 48 h (21). The secondary outcomes were postoperative ICU length of stay, postoperative length of stay, mechanical ventilation duration (MVD), and incidence of hypoxemia (oxygen partial pressure < 80 mmHg). In this study, nasal tube or mask oxygen therapy was considered a low-level oxygen supply, whereas oxygen storage masks, high-flow oxygen therapy, and noninvasive ventilation provided higher oxygen levels. The endpoint events were patient death, failure to wean from mechanical ventilation (Supplementary Materials: the intraoperative and postoperative airway protection strategy and protocol for ventilator weaning) by the time of discharge, or patient discharge.

### Statistical analysis

Continuous variables were first tested for a normal distribution with the Kolmogorov‒Smirnov test. Continuous variables are expressed as the means ± standard deviations and were compared between groups via the *t*-test if they approximately obeyed a normal distribution; otherwise, they are expressed as medians together with quartiles and compared between groups via the Mann-Whitney test. Categorical variables are presented as numbers (percentages) and were compared between groups via the Pearson chi-square test. Univariate analyses were conducted to estimate the risk factors potentially affecting diaphragm function. Following univariate analysis, multivariate logistic regression analysis was conducted on variables with *p*-values of less than 0.2. The test level was set at 0.05. All analyses were performed with SPSS statistical software.

## Results

### General information

**Figure 2:**
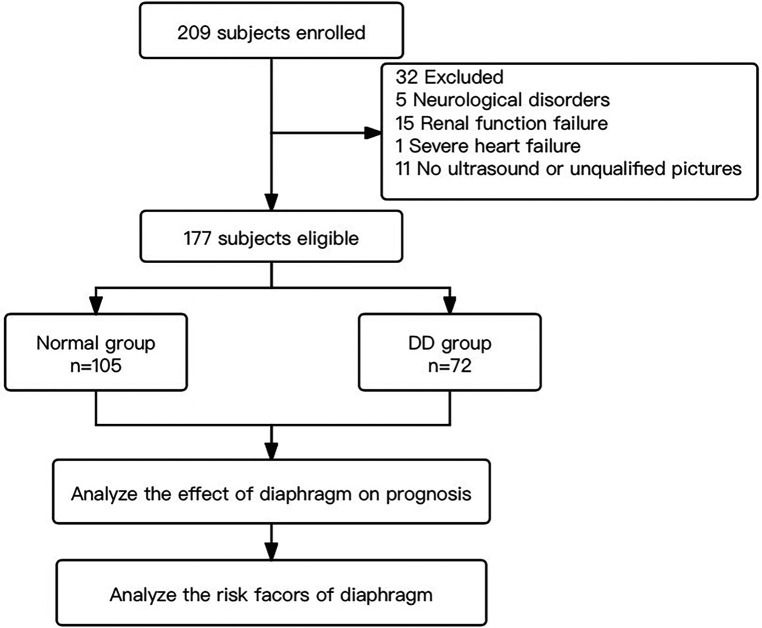
Flow chart of study.

**Figure 3:**
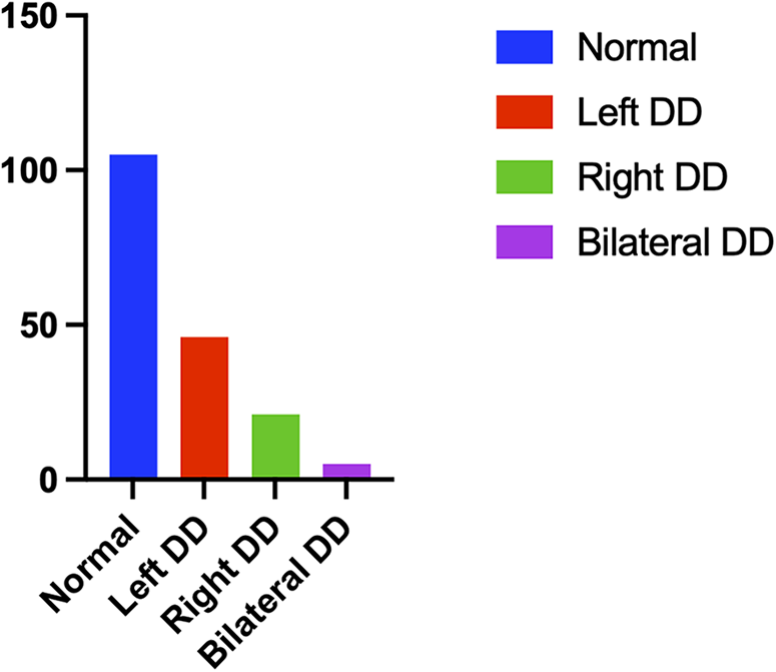
Distribution of diaphragm function of subjects.

From June 2020 to September 2020, a total of 209 eligible patients underwent bedside ultrasound examinations. Thirty-two patients were excluded, 5 of whom had a Glasgow score ≤ 8, hemiplegia or paraplegia, or stroke confirmed by CT examination within 72 h after surgery; 15 of whom were treated with haemofiltration therapy due to severe renal dysfunction within 72 h after surgery; 1 who was treated with extracorporeal membrane oxygenation for severe cardiac dysfunction within 24 h after surgery; and 11 of whom did not undergo diaphragmatic ultrasound or whose only unilateral diaphragm was observed within 24–48 h after surgery (Figure 2). In total, 177 patients were eligible, including 105 patients in the normal group (59 males and 46 females), with an average age of 59.3 ± 1.2 years. The group with diaphragmatic dysfunction comprised 72 patients, representing 40.7% of the enrolled patients—43 males and 29 females—with an average age of 57.4 ± 2.0 years. Among individuals with normal diaphragm function, 82 presented a diaphragm thickness fraction of less than 20% with normal mobility, accounting for 46.3% of the total population. Two individuals exhibited abnormal diaphragm activity, yet their thickness fraction was within the normal range, representing 1.1%. Among all the individuals with diaphragmatic dysfunction, 46 (26.0%) were on the left side, 21 (11.9%) were on the right side, and 5 (2.8%) were on both sides (Figure 3). None of the patients in this study died during the observed period.

There were no significant differences in the distributions of sex, age, or body mass index between the two groups. No significant differences were observed in preoperative data, such as hypertension, diabetes mellitus, preoperative cardiac function (NYHA grade), intraoperative information (such as extracorporeal circulation time), mechanical ventilation time, operation type, and postoperative information (such as postoperative LVEF, SOFA score on the first day after operation, or CRP within 24 h after operation) (Table 1).

**Table 1:** Baseline characteristics of patients.

Variables	Normal group	DD group	*x*^2^/*z*/*t* value	*P* value
General information				
Male (*n*, %)	59 (56.2)	43 (59.7)	0.218	0.640^a^
Age [median (IQR), years]	60 (51, 75)	60 (50, 70)	−0.321	0.748^b^
BMI [median (IQR), kg/m^2^]	24.4 (22.1, 26.6)	23.8 (21.8, 26.1)	−0.642	0.521^b^
Hypertension (n,%)	41 (41.0)	25 (34.7)	0.701	0.403^a^
Diabetes (n,%)	7 (6.7)	10 (13.9)	2.498	0.114^a^
Preoperative NYHA classification (n,%)				
I-II	61 (58.1)	47 (65.3)		
III	40 (38.1)	23 (31.9)	0.949	0.622^a^
IV	4 (3.8)	2 (2.8)		
Surgery factors				
CPB time(mean ± sd, h)	136.0 ± 7.3	135.7 ± 11.6	−0.822	0.412^c^
Surgical procedure (n,%)				
Valve replacement	49 (57.6)	34 (54.8)		
Cardiac bypass	12 (14.1)	14 (22.6)	1.945	0.378^a^
Ascending aorta surgery	24 (28.2)	14 (22.6)		
Postoperative factors				
LVEF (n,%)	55 (50, 58)	56 (52, 59)	−0.821	0.412^b^
Sofa score[median(IQR)]	8 (6, 9)	8 (7, 9)	−0.878	0.380^b^
CRP[median(IQR), mg/l]	157.6 (136.3, 186.9)	162.4 (135.7, 198.4)	−0.077	0.938^b^

DD, diaphragmatic dysfunction; IQR, interquartile range; BMI, body mass index; NYHA, New York Heart Association; CPB, cardiopulmonary bypass; sd, standard devation; LVEF, left ventricular ejection fraction; Sofa score, sequential organ failure assessment score; CRP, C-reactive protein.

^a^ Chi-square test.

^b^ Mann-Whitney test.

^c^ Independent samples *t*-test.

### Effect of diaphragm dysfunction on prognosis

**Figure 4:**
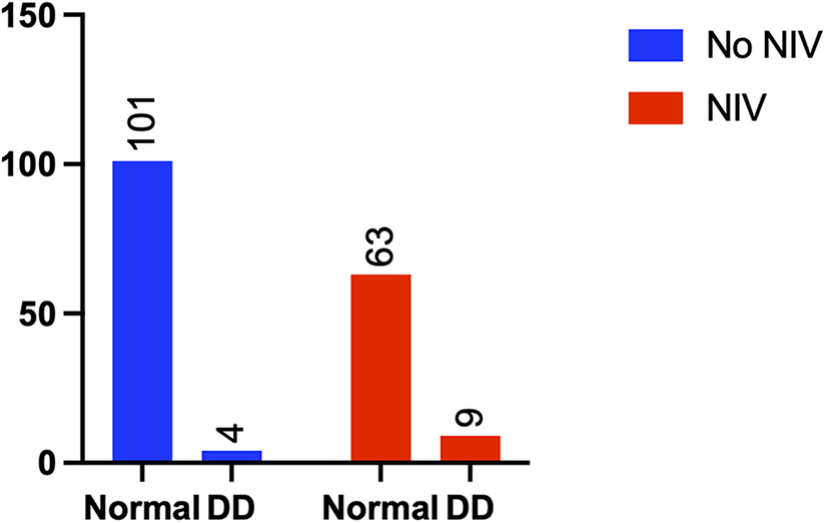
Comparison of NIV between the two groups.

**Figure 5:**
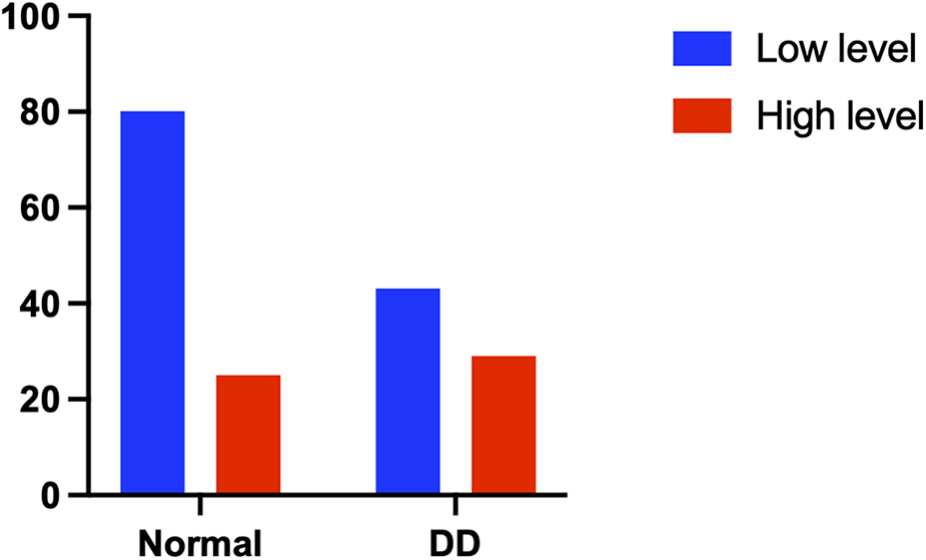
Comparison of oxygen supply between the two groups.

Patients in the diaphragm dysfunction group were more likely to be administered sequential noninvasive ventilation (NIV) within 24 h of weaning (3.8% vs. 12.5%, *p* = 0.029) (Figure 4) and required more oxygen support postweaning (23.8% vs. 40.3%, *p* = 0.019) (Figure 5) than were those in the normal diaphragm function group. Compared with the normal group, the diaphragm dysfunction group presented an increasing trend (*p* = 0.119, *p* = 0.073) in the postoperative ICU or hospital stay. There was no significant difference in the duration of mechanical ventilation or incidence of postoperative hypoxemia between the two groups. No significant differences were found in tracheostomies or the need for reintubation within 48 h of weaning between the two groups (refer to Table 2 for details).

**Table 2:** Outcomes of diaphragm dysfunction on the prognosis.

Variables	Normal group	DD group	*x*^2^/*z* value	*P* value
NIV (*n*, %)	4 (3.8)	9 (12.5)	4.740^b^	**0**.**029**
Tracheostomy (*n*, %)	1 (1.0)	1 (1.4)	0.073^b^	0.787
Reintubation (*n*, %)	3 (2.9)	4 (5.6)	0.819^b^	0.366
Higher level oxygen therapy (*n*, %)	25 (23.8)	29 (40.3)	5.464^b^	**0**.**019**
ICU stay [median (IQR), d]	3.0 (2.0, 4.0)	3.0 (2.0, 6.0)	−1.561^a^	0.119
Postoperative stay [median (IQR), d]	14.0 (11.0, 17.0)	15.0 (11.0, 21.0)	−1.793^a^	0.073
MVD >24 h (*n*,%)	37 (35.2)	20 (27.8)	1.089^b^	0.297
Hypoxemia (n,%)	66 (62.9)	43 (59.7)	0.177^b^	0.674

DD, diaphragmatic dysfunction; NIV, noninvasive ventilation; IQR, interquartile range; MVD, mechanical ventilation duration.

^a^ Mann-Whitney test.

^b^ Chi-square test.

Bold values indicate statistical significance.

### Risk factors for diaphragmatic dysfunction

**Figure 6:**
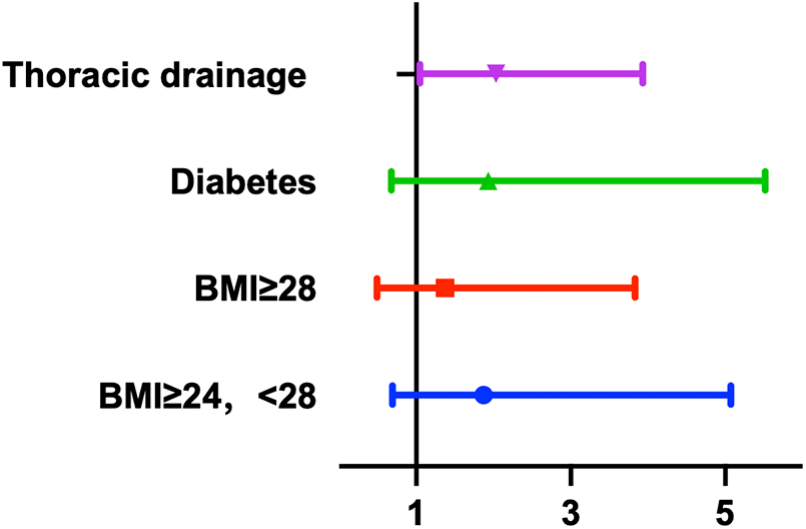
Multivariate regression analysis demonstrated the risk factors of the diaphragm dysfunction.

The placement of a thoracic drainage tube during surgery accompanied by bloody drainage fluid after surgery was closely associated with the occurrence of diaphragmatic dysfunction according to both univariate and multivariate analyses and was an independent risk factor for postoperative diaphragmatic dysfunction (univariate analysis: 95% CI: 1.126–4.137, *p* = 0.021; multivariate analysis: 95% CI: 1.036–3.897, *p* = 0.03). Figure 6 shows the results of the multivariate regression analysis. Our study revealed that preoperative factors, such as sex, advanced age (age >70 years), body mass index, hypertension status, diabetes status, and preoperative cardiac function (NYHA classification); intraoperative factors, such as cardiopulmonary bypass time, type of surgery, blood loss, and volume of transfusion; and postoperative factors, such as the postoperative SOFA score, duration of mechanical ventilation (MVD), postoperative cardiac dysfunction (LVEF < 50), CRP level, sedatives and analgesics within 24 h postoperatively were not significantly associated with the occurrence of diaphragmatic dysfunction (*p* > 0.05). The occurrence of cardiac arrest during coronary artery bypass surgery was not significantly related to postoperative diaphragmatic dysfunction (*p* = 0.05). The detailed data are shown in Table 3.

**Table 3:** Risk factors for diaphragmatic dysfunction.

	Normal group	DD group	Univariate analysis		Multivariate logistic analysis^a^	
			OR (95% CI)	*P* value	OR (95% CI)	*P* value
General information						
Male (*n*, %)	59 (56.2)	43 (59.7)	0.865 (0.471–1.590)	0.641		
Age ≥70 (years)	23 (21.9)	14 (19.4)	1.162 (0.552–2.447)	0.631		
BMI (kg/m^2^)				0.198		0.324
<24 (*n*, %)	45 (42.9)	38 (52.8)	1		1	
24–28 (*n*, %)	42 (40.0)	28 (38.9)	2.533 (0.914–7.024)	0.074	2.141 (0.755–6.069)	0.152
≥28 (*n*, %)	18 (17.1)	6 (8.3)	2.000 (0.707–5.660)	0.192	1.621 (0.557–4.720)	0.376
Hypertension (*n*, %)	41 (41.0)	25 (34.7)	1.304 (0.700–2.428)	0.403		
Diabetes (*n*, %)	7 (6.7)	10 (13.9)	2.235 (0.808–6.180)	0.121	0.528 (0.185–1.503)	0.231
Preoperative NYHA classification (*n*, %)				0.623		
I-II	61 (58.1)	47 (65.3)	1			
III	40 (38.1)	23 (31.9)	1.541 (0.271–8.775)	0.626		
IV	4 (3.8)	2 (2.8)	1.150 (0.195–6.773)	0.877		
Surgery factors						
CPB time (mean ± sd, h)	136.0 ± 7.3	135.7 ± 11.6	1.000 (0.996–1.003)	0.867		
Off-pump heart bypass surgery (*n*, %)	11 (55.0)	8 (47.1)	0.727 (0.199–2.661)	0.630		
Surgical procedure (*n*, %)				0.383		
Valve replacement (*n*, %)	49 (57.6)	34 (54.8)	1			
Cardiac bypass (*n*, %)	12 (14.1)	14 (22.6)	1.190 (0.539, 2.624)	0.667		
Ascending aorta surgery (*n*, %)	24 (28.2)	14 (22.6)	2.000 (0.725, 5.515)	0.180		
Blood loss [median (IQR), ml]	900 (600, 1,425)	950 (687.5, 1,425)	1.000 (1.000–1.000)	0.833		
Blood transfusion [median (IQR), ml]	625 (0, 1,437.5)	500 (0, 1,137.5)	1.000 (1.000–1.000)	0.533		
Postoperative factors						
Sofa score [median (IQR)]	8 (6,9)	8 (7,9)	1.030 (0.899–1.179)	0.674		
MVD >24 h (*n*, %)	37 (35.2)	20 (27.8)	1.415 (0.736–2.718)	0.298		
LEVF <50 (*n*, %)	21 (20.2)	15 (20.8)	1.040 (0.495–2.187)	0.917		
CRP [median (IQR), mg/L]	157.6 (136.3, 186.9)	162.4 (135.7, 198.4)	1.001 (0.996–1.007)	0.646		
Sedatives (*n*, %)	52 (49.5)	39 (54.2)	0.830 (0.455–1.514)	0.544		
Opioids (*n*, %)	23 (21.9)	20 (27.8)	0.729 (0.365–1.458)	0.372		
Thoracic drainage tube accompanied by bloody drainage fluid (*n*, %)	25 (23.8)	29 (40.3)	**2.158** (**1.126–4.137)**	**0**.**021**	2.010 (1.036–3.897)	**0**.**039**

DD, diaphragmatic dysfunction; OR, odds ratio; IQR, interquartile range; BMI, body mass index; NYHA, New York Heart Association; CPB, cardiopulmonary bypass; sd, standard devation; MVD, mechanical ventilation duration; LVEF, left ventricular ejection fraction; Sofa score, sequential organ failure assessment score; CRP, C-reactive protein.

^a^ Variables included in the multivariate regression model were diabetes. BMI, and thoracic drainage tube accompanied by bloody drainage fluid.

Bold values indicate statistical significance.Bold values indicate statistical significance.

## Discussion

In this study, bedside diaphragmatic ultrasonography was used to assess the occurrence of diaphragmatic dysfunction after cardiac surgery and to investigate its impact on patient prognosis and potential risk factors. We found that the incidence of early postoperative diaphragmatic dysfunction after cardiac surgery was 40.7%, with 26.0% left-sided, 11.9% right-sided, and 2.8% bilateral. Patients with diaphragmatic dysfunction have an increased rate of noninvasive ventilation use within 24 h of weaning and require advanced oxygen support (high-flow oxygen inhalation, oxygen storage mask, noninvasive ventilation) after weaning. Univariate and multivariate regression analyses indicated that the placement of a thoracic drainage tube during surgery, accompanied by bloody drainage after surgery, was an independent risk factor for diaphragmatic dysfunction. The high incidence of early postoperative diaphragmatic dysfunction significantly affects respiratory function; thus, the risk factors for this condition should be carefully monitored.

The diaphragm serves as the primary muscle for inspiration, and dysfunction of the diaphragm can lead to restrictive ventilatory disorders and decreased exercise endurance (11). Diaphragmatic dysfunction following cardiac surgery may increase the risk of pneumonia, pulmonary atelectasis, reintubation, tracheotomy, and noninvasive ventilation, thereby extending the duration of mechanical ventilation and ICU hospitalization (4, 5, 8, 9, 22). Previous studies have reported that the incidence rate of diaphragmatic dysfunction after cardiac surgery detected by bedside ultrasound is as high as 70.8%. It is recommended that bedside ultrasound be used as a routine clinical evaluation method for early postoperative rehabilitation and follow-up (23). Dimopoulou et al. reported no significant differences in the duration of mechanical ventilation, ICU stay, or postoperative hospital stay between patients with unilateral diaphragmatic dysfunction and controls (13). In this study, no significant difference was observed in the duration of mechanical ventilation between the group with diaphragm dysfunction and the group with normal diaphragm function. This could be attributed to most of the study participants having unilateral diaphragm insufficiency, constituting 93% of all patients with diaphragm insufficiency. We observed that patients with diaphragm dysfunction required more oxygen support after weaning from mechanical ventilation within 48 h postsurgery, and the proportion of patients who transitioned to noninvasive ventilation postweaning was significantly greater. This research suggested that diaphragmatic dysfunction was significantly associated with postoperative pulmonary complications, similar to the findings of Nørskov's study (24). Although the differences were not statistically significant, patients with diaphragm dysfunction tended to have longer postoperative ICU and hospital stays than patients in the control group.

The risk factors for diaphragm function injury after cardiac surgery are still under exploration. Possible risk factors include hypertension, high BMI, pain, pleural or pericardial effusion, the use of ice to protect the myocardium during surgery, intraoperative blood transfusion, phrenic nerve injury during internal mammary artery isolation, high oxidative stress response, coronary bypass surgery, cardiopulmonary bypass duration, and multiple organ failure (3, 8, 9). There is some controversy surrounding the discussion of certain risk factors. Some scholars believe that separation of the internal mammary artery during cardiac bypass surgery is likely to cause phrenic nerve injury; therefore, the incidence of diaphragmatic dysfunction after cardiac bypass surgery is greater than that after other cardiac surgeries. However, in recent years, due to improvements in surgical techniques, the risk of phrenic nerve injury caused by separation of the internal mammary artery during bypass surgery has gradually decreased (8). In Bruni's study, the incidence rate of diaphragmatic dysfunction in patients who underwent valve replacement surgery was greater than that in patients who underwent cardiac bypass surgery. Moreover, cardioplegia does not increase the incidence of diaphragmatic dysfunction (9). Our findings revealed no significant correlations between sex, age, body mass index, type of cardiac surgery, cardiopulmonary bypass time, SOFA score, or diaphragm dysfunction, which is consistent with the findings of Tralhao et al. and Dimopoulou (12, 13). In our study, postoperative left ventricular dysfunction did not significantly increase the incidence of diaphragm dysfunction, corroborating the findings of Laghlam (19). Dimopoulou suggested that using ice for myocardial protection during surgery was an independent risk factor for diaphragm dysfunction (13). However, in our study, compared with that in patients who underwent off-pump coronary bypass grafting, the incidence of diaphragmatic dysfunction in patients who underwent circulatory arrest coronary artery bypass surgery was not significantly greater, indicating that neither the intraoperative use of cardioplegia nor the intraoperative use of ice to protect the myocardium were risk factors for diaphragmatic dysfunction. Since this study focused on the onset of early diaphragmatic dysfunction (24–48 h postsurgery), the impact of delayed extubation (MVD > 24) on the diaphragm was not entirely apparent. Notably, preserving the integrity of the pleural cavity during thoracotomy is crucial. Rezk et al. discovered that patients who maintained the integrity of the pleural cavity during internal mammary artery transplantation in cardiac bypass surgery experienced a lower risk of postoperative atelectasis and pleural effusion (15). Spadaro et al. reported that lung cancer patients postsurgery in the thoracoscopic surgery group had a lower incidence of diaphragm dysfunction than those in the conventional thoracotomy group (22). In this study, the prevalence of diaphragmatic dysfunction following cardiac thoracotomy was examined, and we identified, for the first time, that the placement of a thoracic drainage tube during surgery accompanied by the presence of bloody postoperative drainage was an independent risk factor for diaphragmatic dysfunction. This finding suggested that breaching the integrity of the pleural cavity during surgery, along with an accompanying postoperative inflammatory response, elevated the risk of postoperative diaphragmatic dysfunction.

The above studies on the risk factors and prognosis of diaphragmatic dysfunction have yielded inconsistent results. Several factors may have contributed to bias in these findings. First, echocardiographic findings in postcardiac surgery patients are compromised by factors such as free gas beneath the xiphoid process and surgical dressings, which can make it challenging to obtain a satisfactory acoustic window. Consequently, in some patients, bilateral diaphragmatic motion may not be acquired (3). To assess the mobility of the left diaphragm, one must observe it through the spleen, which might make it difficult to see the diaphragm. Therefore, some conclusions drawn from research are based on the insufficiency of right-sided function (23). Third, the breathing pattern for bedside diaphragmatic ultrasonography varies among patients who breathe calmly or forcefully or sniff (12, 19, 25). After cardiac surgery, due to sternotomy, pain, and other reasons, patients may be unable to breathe or sniff as hard as they should, resulting in deviations in the results.

Specific treatments can facilitate the recovery of diaphragmatic function. For example, administering bilateral phrenic nerve electrical stimulation for 2 h every 8 h for 48 h can lead to a 15% increase in diaphragmatic thickness (16). A protective ventilation strategy for the diaphragm can help reverse the process of diaphragm atrophy in assisted mode (17), and inspiratory muscle training can enhance the functionality of the diaphragm (18, 26). For patients with persistent diaphragmatic disorders, diaphragmatic plication surgery may also be considered (26).

The limitations of this study were as follows. First, diaphragmatic screening was conducted on the second day after surgery, without continuous follow-up, to monitor the recovery of patients with diaphragmatic dysfunction. Second, when the left diaphragm was screened, some patients’ diaphragms were not clearly visible due to the acoustic window, particularly in patients suspected of having diaphragmatic paralysis, where no significant diaphragm movement was detected according to excursion measurements. This might have resulted in the exclusion of some patients with diaphragmatic dysfunction, leading to biased results. Third, for patients with diaphragmatic paralysis, identifying the beginning of the respiratory cycle at rest is challenging (27). In addition, even healthy individuals may exhibit abnormal diaphragmatic thickening at rest (28). Moreover, even at rest, the activity of the diaphragm can be influenced by sternotomy or pain. Consequently, when only breathing at rest is analyzed, abnormal movements might be confused with normal activity, resulting in a misinterpretation of diaphragmatic function. Finally, the study did not include a transdiaphragmatic pressure measurement, which is the reference test used to assess diaphragm function. We did not perform the analysis of the biomarkers able to identify diaphragm dysfunction due to the retrospective analysis (29).

On the basis of the findings of this study, we recommend the use of diaphragmatic ultrasound to screen high-risk individuals for diaphragmatic dysfunction following cardiac surgery. Early recognition of diaphragmatic dysfunction and early intervention are beneficial for improving the prognosis of patients.

## Conclusion

Early diaphragmatic dysfunction assessed by ultrasonography after cardiac surgery increases the likelihood of requiring sequential noninvasive ventilation upon weaning, necessitates higher levels of oxygen support, and tends to increase postoperative ICU and hospital stays, calling for clinical vigilance and early intervention. A high incidence of diaphragmatic dysfunction was observed in the presence of chest tube placement accompanied by bloody drainage.

## Data Availability

The raw data supporting the conclusions of this article will be made available by the authors, without undue reservation.
