# Supplementary material for: Early diaphragm dysfunction assessed by ultrasonography after cardiac surgery: a retrospective cohort study
Source: Front Cardiovasc Med. 2024 Oct 9;11:1457412. doi: 10.3389/fcvm.2024.1457412 (PMC11496164; doi:10.3389/fcvm.2024.1457412)

The intraoperative and postoperative airway protection strategy and protocol for ventilator weaning in Post-Cardiac Surgery Patients

The intraoperative airway protection strategy involves intermittent positive pressure, tidal volume 6 to 10 mL/kg，respiratory rate 12 to 16 breaths per minute，positive end-expiratory pressure (PEEP) 4 to 7 cm H_2O, airway pressure 10-20 cm H_2O, and a fraction of inspired oxygen (FiO_2) of 50% to 100%. During surgery, adjust ventilator settings based on blood gas analysis results to maintain hemodynamic stability (Supplementary Table 1).

Supplementary Table 1 Ventilation Strategies for Intraoperative Airway Protection

1. before and after CPB with intermittent positive pressure；
2. tidal volume 6 to 10 mL/kg (typically 6 to 8 mL/kg)；
3. respiratory rate 12 to 16 breaths per minute；
4. positive end-expiratory pressure (PEEP) 4 to 7 cm H_2O, and a fraction of inspired oxygen (FiO_2) of 50% to 100%，
5. Airway pressure 10-20 cm H2O
6. Improvement in the primary conditions causing respiratory disorders。
7. awake and able to follow simple commands;

The postoperative airway protection strategy involves setting initial ventilator parameters to SIMV mode with a tidal volume of 6-8 mL/kg, plateau pressure (Pplat) less than 30 cmH2O, PEEP at 5 cm H2O, and a respiratory rate of 12-16 breaths per minute. Subsequently, the ventilator settings are adjusted according to blood gas analysis results to ensure hemodynamic stability. Pre-extubation assessment begins 2-3 hours after ICU admission, with essential criteria including hemodynamic stability, homeostasis, absence of electrolyte imbalance, and recovery of spontaneous breathing with PEEP no more than 5 cmH2O and PaO2/FiO2 no less than 150 mmHg, as well as chest fluid drainage less than 100 ml/h and no significant pleural effusion. Individuals who met the pre-extubation assessment criteria were to undergo a Spontaneous Breathing Trial (SBT).

The spontaneous breathing trial (SBT) typically involves 30 minutes of Continuous Positive Airway Pressure (CPAP) combined with minimal pressure support (2-7 cmH2O). Indicators of weaning failure include HR > 140 bpm (or an increase of > 20%) and newly emerged significant arrhythmia, respiratory rate > 35 breaths/minute, systolic blood pressure increases by more than 180 mmHg or decreases by less than 90 mmHg, SpO2 ≤ 90%, PaO2 ≤ 50 mmHg, pH ≤ 7.32 and hyperhidrosis or dysphoria.

After successfully completing the SBT, the endotracheal tube can be removed based on a comprehensive evaluation of muscle strength, airway self-protection ability, consciousness status, and airway secretion volume(Supplementary Table 2 and Supplementary Figure 1)

Supplementary Table 2 Evaluation criteria before ventilator weaning form ventilation

1. hemodynamic stability with minimal vasopressor support or none；No malignant arrhythmia；
2. no significant metabolic acidosis, homeostasis and blood gases within the normal ranges;
3. spontaneous breathing recovered；

4. PEEP less than 5 cmH_2O；PaO_2/FiO_2 greater than 150 mmHg； when FiO_2 less than 40%，SpO_2 greater than 90%
5. Chest fluid drainage less than 100 ml/h; no large pleural effusion;
6. adjust ventilator settings according to blood gas analysis results to ensure hemodynamic stability

Supplementary Figure 1: Protocol for Ventilator Weaning in Post-Cardiac Surgery Patients

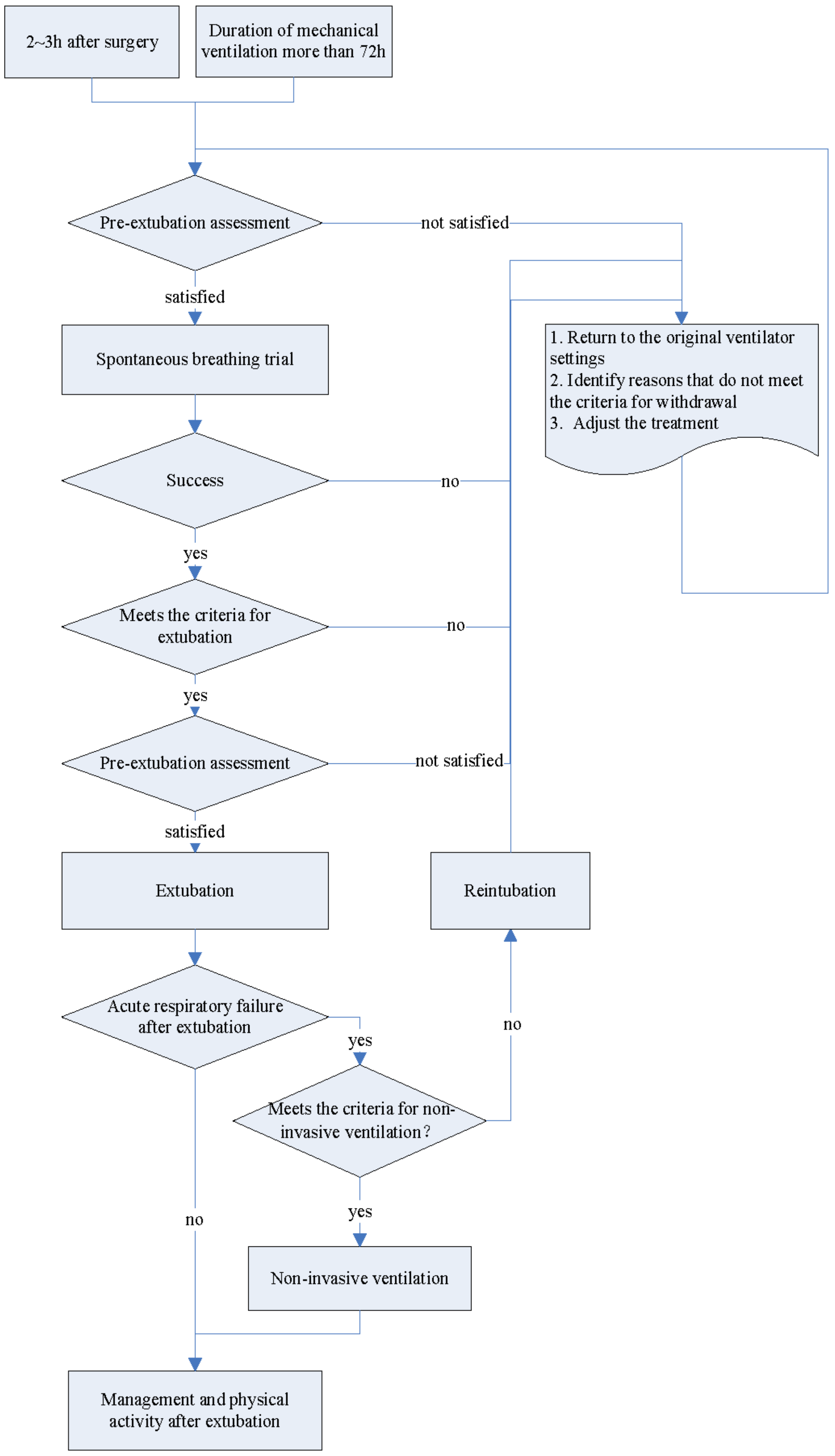

Supplement: Supplementary file 1 [file Datasheet1.pdf]
